# Management of Refractory Chronic Obstructive Pulmonary Disease: A Review

**DOI:** 10.3390/life14050542

**Published:** 2024-04-24

**Authors:** Mandeep Singh Rahi, Mayuri Mudgal, Bharat Kumar Asokar, Prashanth Reddy Yella, Kulothungan Gunasekaran

**Affiliations:** 1 Division of Pulmonary Diseases and Critical Care Medicine, Yale-New Haven Health Lawrence and Memorial Hospital, New London, CT 06320, USA; 2 Department of Internal Medicine, Camden Clark Medical Center, Parkersburg, WV 26101, USA; mayuri.mudgal@wvumedicine.org; 3 Division of Medical Sciences, The Tamilnadu Dr. MGR University, Chennai 600032, Tamilnadu, India; bharatkumarasokar@gmail.com; 4 Department of Internal Medicine, Yuma Regional Medical Center, Yuma, AZ 85364, USA; pryella@yumaregional.org; 5 Division of Pulmonary Diseases and Critical Care Medicine, Yuma Regional Medical Center, Yuma, AZ 85364, USA; kgunasekaran@yumaregional.org

**Keywords:** COPD, refractory COPD, lung volume reduction surgery, biologics in COPD

## Abstract

Chronic obstructive pulmonary disease (COPD) is a common condition with an estimated prevalence of 12% in adults over the age of 30 years worldwide. COPD is a leading cause of morbidity and mortality globally, with a substantial economic and social burden. There are an estimated 3 million deaths annually due to COPD. However, most of the patients with COPD respond to routine interventions like bronchodilator therapy, assessing supplemental oxygen needs, smoking cessation, vaccinations, and pulmonary rehabilitation. There is a significant number of patients who unfortunately progress to have persistent symptoms despite these interventions. Refractory COPD is not yet formally defined. Patients with severe persistent symptoms or exacerbations despite appropriate care can be considered to have refractory COPD. Managing refractory COPD needs a multidimensional approach. In this review article, we will discuss essential interventions like ensuring adequate inhaler techniques, exploring the need for non-invasive ventilatory support, use of chronic antibiotics and phosphodiesterase inhibitors to advanced therapies like bronchoscopic lung volume reduction surgery, and the upcoming role of anti-IL5 agents in managing patients with refractory COPD. We will also discuss non-pharmacologic interventions like psycho-social support and nutritional support. We will conclude by discussing the palliative care aspect of managing patients with refractory COPD. Through this review article, we aim to better the approach to managing patients with refractory COPD and discuss new upcoming therapies.

## 1. Introduction

Chronic obstructive pulmonary disease (COPD) is a heterogeneous lung condition affecting the airways (chronic bronchitis) and alveoli (emphysema) that causes persistent and often progressive symptoms of dyspnea, cough, and sputum production [1]. In addition to tobacco smoking, exposure to toxic particles and gases from household and outdoor air pollution also leads to COPD [2]. Host factors include alpha-1-antitrypsin deficiency, abnormal lung development, and accelerated lung aging [3,4]. The COPD prevalence data vary widely due to differences in survey methods, diagnostic criteria, and statistical approaches [5]. Based on the Burden of Obstructive Lung Disease (BOLD) program and other large-scale epidemiological studies, the global prevalence of COPD is estimated to be around 10.3% (95% confidence interval (CI) 8.2%,12.8%) [6,7]. According to the Centers for Disease Control and Prevention (CDC), among US adults from 2011 to 2020, COPD was more prevalent among women compared with men. COPD is one of the leading causes of morbidity and mortality worldwide, with a substantial economic and social burden [8]. COPD morbidity and mortality vary across countries [9]. COPD was the sixth leading cause of death in the United States of America in 2020 and, globally, there are around three million deaths annually due to COPD [10,11]. Typical symptoms of COPD include dyspnea, wheezing, chest tightness, fatigue, and cough, which could be productive and may experience acute events or increased respiratory symptoms called exacerbations [12]. Standard interventions like bronchodilator therapy, vaccinations, smoking cessation, and pulmonary rehabilitation can control the majority of patients with less severe COPD [13,14]. There is no universal definition of refractory COPD. Patients with severe progressive and persistent symptoms and recurrent exacerbations despite standard medical interventions could be considered to have refractory COPD. Patients deemed to have refractory COPD need a multidisciplinary approach and consideration of advanced medical and surgical therapies with the primary goal of alleviating symptoms and improving quality of life. These patients commonly have other medical co-morbidities like cardiac disease, arrhythmias, peripheral vascular disease, anxiety and depression, diabetes mellitus, and liver diseases that need to be recognized and managed promptly [15,16]. This article discusses the assessment and management of patients with refractory COPD.

## 2. Initial Assessment of Patients with Refractory COPD

### 2.1. Evaluation of Dyspnea, Exacerbation History, and Smoking History

In patients considered to have refractory COPD, the diagnosis of COPD should be confirmed by reviewing their prior pulmonary function testing (PFT) and observing the trend in PFTs. Dyspnea and exacerbation history should be quantified using the Global Initiative for Chronic Obstructive Lung Disease (GOLD) guidelines [17]. Dyspnea could be quantified using validated tools such as the modified Medical Research Council (mMRC) dyspnea scale or the COPD Assessment Test (CAT). Detailed exacerbation history should be assessed, including the frequency (any treatments with antibiotic and corticosteroids in an outpatient or inpatient setting) and severity (needing hospitalization, intensive care unit stay, non-invasive positive pressure ventilation, or invasive mechanical ventilation). Baseline symptoms like dyspnea severity, exertional ability, cough frequency, sputum volume, and purulence should also be established. In addition to a detailed smoking history, marijuana use and vaping usage should be documented as well [18,19]. Lastly, if not already achieved, smoking cessation is essential to prevent further decline in lung function, and various pharmacological and non-pharmacological approaches are available to accomplish this [20].

### 2.2. Optimizing Inhaler Technique

It is assumed that patients with refractory COPD have persistent symptoms despite maximal inhaler therapy with long-acting beta-agonist (LABA), long-acting antimuscarinic (LAMA), and inhaled corticosteroids (ICS). Multiple inhaler types (MDI (metered dose inhaler), DPI (dry powder inhaler), SMI (soft mist inhaler or nebulizer)) and dosing schedules could lead to inconsistent or improper use. Most COPD patients use inhalers, but specific patient populations, like those with cognitive, neuromuscular, or ventilatory impairment and those with suboptimal peak inspiratory flow, may not derive full benefit from inhalers and require nebulization for drug delivery [21]. Interestingly, in large population studies, inhaler adherence only ranges from 20 to 50%, and two-thirds of patients with asthma or COPD have improper inhaler use [22,23,24]. This leads to frequent hospitalization and a more significant economic burden [25]. Therefore, lack of adherence is an essential challenge in managing patients with COPD. Barriers to adherence should be explored, such as complex regimens with multiple inhaler types, language barriers, lack of regular access to medication, cost of inhalers, stress, depression, and lack of confidence in the technique [26,27]. Attempts to simplify the inhaler regimen and ensure proper inhaler usage (using patient education resources and supervised usage during follow-up visits) should be regularly carried out.

### 2.3. Pulmonary Function Testing, Arterial Blood Gas Analysis, and Cardiothoracic Imaging

Patients with refractory COPD should have repeat PFTs to re-evaluate airflow, lung volumes, and gas exchange. Worsening obstruction with increased air trapping and hyperinflation suggests worsening obstruction, and patients could be considered for lung volume reduction surgery. If a new restrictive process or diffusion impairment is identified, prompt chest imaging and transthoracic echocardiogram (TTE) should be performed to rule out interstitial lung disease and pulmonary hypertension or cor pulmonale, respectively. Patients should have arterial blood gases (ABG) analysis to look for uncompensated hypercapnia (to determine the need for nocturnal non-invasive ventilation) and hypoxemia (to determine the need for long-term oxygen therapy (LTOT). Similarly, patients should have exercise pulse oximetry to determine the need for LTOT. Repeat CT imaging should be considered to exclude co-morbidities like ILD, bronchiectasis, bronchiolitis, central airway obstruction, evidence of pulmonary edema or pulmonary hypertension, and lung cancer.

### 2.4. Evaluating Co-Morbidities

Patients with COPD are likely to have co-morbidities that frequently contribute to dyspnea and exercise impairment. Some of the significant concomitant cardiovascular diseases include coronary artery disease (CAD), congestive heart failure (CHF), especially right heart failure or cor pulmonale, cardiac arrhythmia, and pulmonary hypertension. Other common co-morbidities include sleep-disordered breathing, obesity/metabolic syndrome, diabetes, anxiety, and depression [15,16]. These conditions should be assessed with a thorough medical history and physical examination. A low threshold should be maintained to obtain additional testing like transthoracic echocardiogram, cardiac stress test, polysomnogram, and computed tomography of the chest to identify and treat some of these common co-morbidities.

## 3. Non-Pharmacological Approaches for Refractory COPD Management

### 3.1. Nutrition

Even with current therapy, up to 60% of patients with refractory COPD still experience severe weight loss or lean body mass loss due to malnutrition [28]. This muscle atrophy and cachexia lead to serious side effects, including an accelerated decline in lung function, lowered immunity, exercise intolerance, and a noticeably higher risk of death [29,30]. Oral nutritional supplements have demonstrated some effectiveness. High-calorie supplements or enteral formulations have been shown to provide some sustained weight gain and reduce fat-free mass depletion, but most of these trials were small or did not include adequate controls [29,31]. Furthermore, more comprehensive functional effects such as increased strength, a higher quality of life connected to health, or lower healthcare utilization are absent. Two prospective trials of rigorous nutritional supplementation combined with resistance training, anabolic steroids, and acute exacerbation therapy revealed significant increases in outcomes, including exercise capacity and survival [32]. Nevertheless, more research is needed to determine the optimal supplement formulation for the hypermetabolic condition of refractory COPD. Certain nutrients, such as probiotic strains, antioxidants, and omega-3 fatty acids, may influence gut integrity, infection risk, and systemic inflammation [33]. Overall, the current research does not support the routine use of dietary therapies to improve long-term prognosis without concurrently addressing underlying COPD disease activity, even though some degree of short-term stabilization appears feasible through these means [29,31]. Randomized controlled studies that directly compare oral supplement formulations and enteral feeding techniques in integrated patient-centered illness care are among future priorities.

### 3.2. Pulmonary Rehabilitation

The American Thoracic Society and European Respiratory Society define pulmonary rehabilitation as “comprehensive intervention based on a thorough patient assessment followed by patient-tailored therapies that include but are not limited to, exercise training, education, and behavior change, designed to improve the physical and psychological condition of people with chronic respiratory disease and to promote the long-term adherence to health-enhancing behaviors” [34]. Pulmonary rehabilitation consists of exercise training (including endurance training, upper extremity, and lower extremity exercises), promotion of healthy behaviors (smoking cessation, regular exercise, healthy nutrition, adherence to and proper use of prescribed medications, and disease self-management), and psychological support (e.g., improving self-efficacy and providing coping strategies for chronic illness) [35]. The Global Initiative for Chronic Obstructive Disease (GOLD) recommends pulmonary rehabilitation as part of an integrated management approach in patients who are symptomatic with functional limitations and patients with recurrent exacerbations (Group B and E). Pre-program evaluation includes assessment of respiratory impairment, exercise tolerance (using 6-min walk tests, shuttle walk test, or cardiopulmonary exercise testing), and presence of co-morbidities, especially cardiac, neurologic, and musculoskeletal. The optimal duration of a pulmonary rehabilitation program is not known; the typical length is 8–12 weeks. Studies have shown that benefits plateau within 12 weeks [34,36]. Pulmonary rehabilitation has been shown to improve exercise capacity and quality of life. A meta-analysis of 65 randomized controlled trials showed clinically significant improvement in the 6-min walk distance when compared with standard care (mean difference (MD) 43.93 m, 95% CI 32.64 to 55.21). Similarly, it showed improved scores in four major domains of quality of life, including dyspnea, fatigue, emotional function, and mastery [37]. Studies suggest a possible mortality benefit, but the quality of evidence is low and inconsistent across the studies [38]. Unfortunately, the benefits of pulmonary rehabilitation decline over time, and the timing and role of retraining have not been defined. However, an observational study suggests maintenance with a self-monitored program is helpful in retaining improvements in exercise tolerance and health status [39].

### 3.3. Non-Invasive Positive Pressure Ventilation

Patients with refractory COPD and daytime hypercapnia or nocturnal hypoxemia not responsive to nocturnal oxygen therapy are potential candidates for non-invasive positive pressure ventilatory (NIPPV) support. COPD patients should be assessed for OSA with polysomnography or referral to a sleep medicine specialist, as this would be treated differently. Patients who have low clinical suspicion of OSA or those with normal polysomnogram but nocturnal hypoxemia should be assessed further for NIPPV candidacy. These patients should undergo repeat PFTs (if not carried out recently) and arterial blood gas (ABG) analysis. In advanced COPD, to overcome the hypercapnia caused by V/Q mismatch, the respiratory muscles must work more to increase the minute ventilation. Due to changes in diaphragm configuration and inadequate nutritional support, respiratory muscle exhaustion is caused by the increased ventilatory load [40]. Using NIPPV in such patients has been hypothesized to improve respiratory function by providing rest to already overworked respiratory muscles, although evidence to support this is rudimentary [41]. Old case series have shown sustained improvement in daytime hypercapnia in patients using nocturnal NIPPV [42]. More recently, a retrospective study showed improvement in spirometry indices, lung hyperinflation, and daytime hypercapnia over one year [43].

Nocturnal NIPPV can be initiated in two of the following clinical settings: Firstly, in patients with advanced COPD and chronic daytime hypercapnia (PaCO_2_ > 52 mm Hg) or nocturnal hypoxemia (SpO_2_ < 88% for >5 min out of >2 h of nocturnal oximetry) despite supplemental oxygen at >2 L/min [44]. Secondly, advanced COPD patients with acute exacerbation. The American Thoracic Society recommends deferring nocturnal NIPPV initiation until after the resolution of acute exacerbation, as around 20% of patients who require NIPPV during hospitalization will no longer be hypercapnic four weeks after discharge [44,45]. There is a consistent signal regarding reduced mortality with NIPPV use. In a meta-analysis with over 800 patients, there was a 14% reduction in mortality in the NIPPV group compared with usual care alone [44]. In contrast, data regarding improving dyspnea, quality of life, and PFTs are mixed. A meta-analysis with over 13 randomized trials showed improved dyspnea scores and exercise tolerance with NIPPV [44]. However, another systematic review with over 200 patients found no benefit for PaCO_2_, health-related quality of life scores (HRQoL), six-minute walk distance (6MWD), and PFT values [46]. Some recent studies examining the efficacy of NIPPV are described in Table 1. The practical challenges of NIPPV vary regionally and depend on the clinical scenario. A bi-level positive airway pressure, or BPAP, is typically used for patients hospitalized for COPD exacerbation. Since these patients are observed closely in the hospital, their optimal settings can be determined [47]. NIPPV initiation in non-hospitalized advanced COPD patients could be cumbersome. Typically, efforts should be made to initiate nocturnal NIPPV in a sleep laboratory or hospitalized setting where the patient can be monitored closely and, at the same time, would help exclude obstructive and central sleep apnea (OSA and CSA) [48,49]. However, the American Thoracic Society recognizes this as a challenge given that sleep laboratories may not be readily available and that reimbursement could be suboptimal. Therefore, ATS gave a conditional recommendation on home initiation over in-lab polysomnography [44]. Physicians work closely with the respiratory therapists and patients to determine the optimal BPAP initiation settings, which typically is with an expiratory positive airway pressure (EPAP) of 5–8 cm of H_2_O and an inspiratory positive airway pressure (IPAP) of 12–20 cm of H_2_O guided by the patient tolerance [44]. Post-initiation follow-up visits should focus on the patient’s acceptance, changes in dyspnea, exercise ability, and symptoms like morning headaches. ABG and PaCO_2_ could help guide the titration [45,50].

### 3.4. Managing Co-Morbidities

COPD often exists with other diseases that can have a significant impact on morbidity and mortality. Cardiovascular diseases (CVDs) are the most common coexisting condition in COPD patients. COPD patients with no prior history of CVD have a 25% increased risk of major adverse cardiac events [54], such as congestive heart failure (CHF), ischemic heart disease, and cardiac arrhythmias. Unrecognized CHF accompanies 40% of patients with acute exacerbation of COPD [55]. There is an increased risk of myocardial infarction within 90 days of an acute exacerbation of COPD. CHF and IHD should be managed according to local and national guidelines in conjunction with a cardiologist. COPD patients should be included in the lung cancer screening program (which includes a low-dose computed tomography scan of the chest) according to local or national guidelines. The United States Preventative Services Task Force (USPSTF) recommends annual low-dose CT scan screening for individuals ages 50 to 80 years with a 20-pack-year history of smoking and current smoker or has given up smoking within the past 15 years [56]. Management of lung cancer should be carried out according to the national guidelines in conjunction with oncologists and thoracic surgeons [57]. About 50% of COPD patients will have some degree of bronchiectasis [58]. Bronchiectasis should be managed per local guidelines, which include airway clearance techniques, early treatment of pseudomonas aeruginosa infection, and cautious use of inhaled corticosteroids (ICS), especially in patients with bacterial colonization or recurrent lower respiratory tract infection [58]. Obstructive sleep apnea (OSA) is lately increasingly diagnosed, with an estimated prevalence of 20% of the US adult population [59]. Concomitant COPD and OSA have a worse prognosis, increased risk of cardiac arrhythmias, and more profound hypoxemia [60]. The use of positive pressure ventilation reduces hospitalization, emergency room visits, and moderate to severe exacerbation episodes [61]. Metabolic syndrome and diabetes are common in patients with COPD and should be managed according to local and national guidelines [15]. Similarly, anxiety and depression are important and underdiagnosed coexisting diseases with COPD that are associated with poor prognosis. It should be assessed periodically in patients with COPD and treated the same for patients without COPD [16].

## 4. Pharmacological Approaches for Refractory COPD Management

Pharmacological options for patients with repeated exacerbations are discussed below despite treatment with long-acting muscarinic agents, long-acting beta-agonists, and inhaled glucocorticoids (ICS) as a combination therapy [17,62]. ICS alone should not be used in patients with COPD. ICS in combination with long-acting bronchodilator therapy could be used in select patients with two or more exacerbations in a year, a history of hospitalization due to exacerbations, a blood eosinophil count of more than 300 cells/microL, or a history of concomitant asthma. ICS therapy should not be used in patients with recurrent pneumonia, mycobacterial infections, bacterial colonization, or a blood eosinophil count less than 100 cells/microL [3].

### 4.1. Long-Term Oxygen Therapy (LTOT)

Long-term oxygen therapy (LTOT) is used for severe hypoxemia (defined as oxygen saturation of ≤88 percent or arterial partial pressure (PaO_2_) of ≤55 mmHg). It is also used for COPD patients with PaO_2_ of 56–59 mmHg or oxygen saturation ≤89% with evidence of end-organ disease (pulmonary hypertension, cor pulmonale, hematocrit > 55%, arrhythmias, congestive heart failure, or impaired mental status). In prior trials, continuous ambulatory supplemental oxygen (unlike nocturnal oxygen therapy) was noted to improve survival, reverse hypoxemia, and decrease pulmonary vascular resistance [63]. The INOX trial studied the use of supplemental oxygen in patients with isolated nocturnal hypoxemia. The trial was underpowered as recruitment was stopped prematurely. This trial did not show any positive or detrimental effect of supplemental oxygen in the selected COPD population [64]. In clinical trials, LTOT in COPD patients with moderate hypoxemia (defined as PaO_2_ >56 mmHg or SpO_2_ level 89–93%) did not demonstrate a survival benefit. It is important to note that, in one of the trials, COPD patients with severe disease with no resting hypoxemia but who were prescribed oxygen therapy had increased mortality and, therefore, detrimental outcomes [63,65]. Similarly, the use of LTOT in exertional hypoxemia has been assessed. Trials have noted improvement with dyspnea and exercise tolerance (likely from improved muscle oxygen delivery, reduced minute ventilation, and decreased pulmonary vascular resistance) but no long-term change in survival outcomes [66]. Data on LTOT use for nocturnal hypoxemia (defined as SpO_2_ < 90% for >30% of the duration on nocturnal oximetry) are limited but note no improvement in mortality.

### 4.2. Chronic Suppressive Antibiotic Therapy

COPD patients have persistent inflammation secondary to chronic bacterial colonization, leading to epithelial damage from airway neutrophilia and interleukin-8 production [67,68]. Therefore, there is a potential role for chronic antibiotic therapy in preventing/reducing COPD exacerbations. Macrolides have been extensively investigated for their immunomodulatory effects. They improve alveolar macrophage phagocytosis, decrease the hypersecretion of pro-inflammatory cytokines and chemokines, maintain the integrity of the airway epithelium, and decrease bacterial colonization, lowering systemic inflammation. Therefore, they are essential in managing COPD [69,70,71,72,73].

There have been changing views regarding the use of continuous antibiotics to decrease the frequency of exacerbations in patients with COPD. Earlier studies showed no effect on exacerbations but noted decreased lost working days [74,75]. Interestingly, these antibiotics belonged to the antibacterial classes of tetracyclines and penicillins. Later, macrolides and fourth generation fluoroquinolones were assessed, and the continuous use of macrolides was noted to have significant benefits in reducing exacerbation in patients with COPD [76]. Moreover, it was pointed out that both daily and intermittent (at least three times a week) macrolide dosing reduced the exacerbation rate [77,78]. Fluoroquinolones, namely moxifloxacin, have also demonstrated a decreased risk of COPD exacerbation. Still, they have higher side effect profiles and a higher emergence of antibiotic resistance bacteria; therefore, their use for treating infections is suggested [79,80]. Macrolides can lead to cardiac arrhythmias. Although the incidence is low (1 in 100,000), it is increased with other co-existing risk factors [81]. Other adverse effects include diarrhea and reversible hearing decrement [82].

### 4.3. Phosphodiesterase Inhibitors

Cyclic-3′,5′-adenosine monophosphate (cAMP)-specific phosphodiesterase (PDE-4) is expressed in multiple tissues in the body, including airway smooth muscles, and metabolizes cAMP into adenosine monophosphate [83]. Therefore, the inhibition of PDE4 leads to increased cAMP levels, which are responsible for relaxing the smooth muscles of the airway and reducing inflammatory markers and cell chemotaxis. Roflumilast is a selective PDE4 inhibitor that reduces the risk of exacerbation in patients with severe COPD with a known history of frequent exacerbation [84,85,86].

Many randomized placebo-controlled trials have been performed evaluating the PDE4 inhibitor effect on lung function and COPD exacerbations in patients with severe COPD. Data from one of the systematic reviews noted reduced chances of exacerbation and mild improvement in forced expiratory volume in one second (FEV1) but little impact on breathlessness and quality of life [87]. Similarly, another trial, Roflumilast and Exacerbations in Patients Receiving Appropriate Combination Therapy (REACT), compared roflumilast to a placebo in COPD patients using inhaler combination standard therapy and noted reduced exacerbations and hospital admissions [88]. Roflumilast use has been known to cause gastrointestinal adverse effects, namely diarrhea, weight loss, and nausea, as well as psychiatric effects, including anxiety, depression, insomnia, and suicidal ideation [86,89]. Since these adverse effects are thought to be dose-dependent, treatment should be initiated with 250 mcg once daily for four weeks and increasing gradually to 500 mcg daily to ease the adverse gastrointestinal effects [90].

Theophylline is a non-selective phosphodiesterase inhibitor with a long history of use in COPD patients. A meta-analysis that included 20 randomized trials showed improvement in FEV1 with theophylline compared with placebo [91]. Theophylline has a narrow therapeutic window with multiple adverse effects (headache, nausea, seizures, and cardiac arrhythmias). It is not commonly used in the developed world but still might be an option in developing countries.

### 4.4. Chronic Glucocorticoids

Systemic corticosteroids have shown a small beneficial effect with a reduction in relapse of COPD exacerbation in symptomatic patients. Many studies have shown that a blood eosinophil level greater than 2% predicts a helpful response to corticosteroid use in this patient population [92,93,94]. Their chronic use for refractory COPD has not shown any evidence of prevention/reduction of exacerbations and, therefore, is not recommended due to the significant detrimental impact on morbidity and mortality [95,96,97]. Moreover, adverse effects, namely steroid myopathy, osteoporosis, and increased risk of pneumonia, are substantial [98,99].

### 4.5. Role of Biologics in COPD

A subset of patients with COPD who continue to experience exacerbations despite being on standard-of-care therapy and chronic antibiotic/PDE-4 inhibitor therapy can benefit from the blockade of the cytokines (IL-4, IL-5, IL-13) involved in type 2 inflammation. Though it is well known that COPD is a neutrophilic predominant inflammation, sputum cellular counts have determined other COPD phenotypes, namely eosinophilic, mixed eosinophilic, and pauci-granulocytic [100]. It is noted that 40% of COPD patients have eosinophilic inflammation [101]. Monoclonal antibody therapies directed against interleukins-4, -5, and -13 are discussed below.

### 4.6. Anti-Interleukin-5/Interleukin-5 Receptor Monoclonal Antibody

IL-5 is a cytokine responsible for eosinophile differentiation, maturation, recruitment, and degranulation [102]. Two monoclonal antibodies directed against IL-5 and IL-5 receptors, namely mepolizumab and benralizumab, respectively, have been studied. A systematic review and meta-analysis noted that mepolizumab reduced the rate of moderate to severe COPD exacerbation by 19% (rate ratio 0.82, 95% CI 0.68–0.98) in patients with an eosinophil count more than 150/microL compared with a placebo. Benralizumab reduced the rate of severe exacerbation only in a subset of patients with an eosinophil count of 220/microL and three or more COPD exacerbations in the last year, with moderate certainty evidence [103]. Also, this result was obtained with the highest dose of this medication at 100 mg every eight weeks.

### 4.7. Anti-Interleukin-4 Monoclonal Antibody

**Figure 1:**
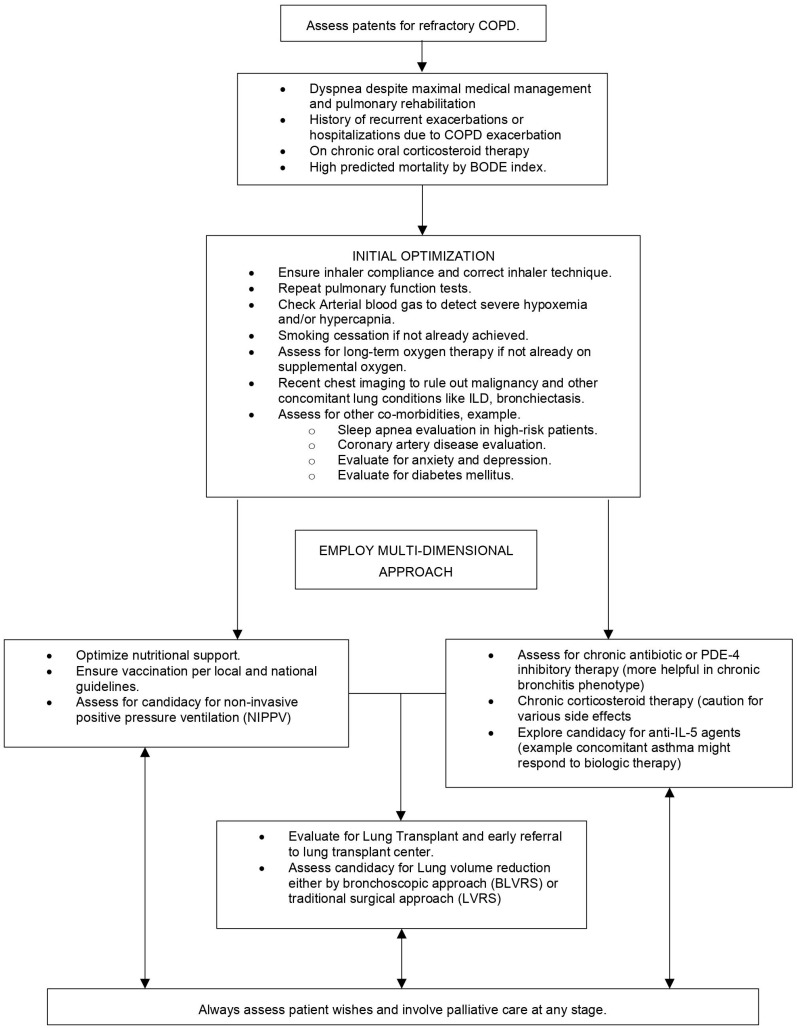
Overviews assessing and managing patients with refractory chronic obstructive pulmonary disease.

IL-4 and IL-13 cytokines majorly contribute to inflammation, causing upregulation of T2 immune response, switching B cell immunoglobulin to IgE and IgG4, stimulating eosinophil migration to sites of inflammation, and increasing airway contractility [104,105]. Dupilumab is a human monoclonal antibody that blocks the common receptor for interleukin-4 and interleukin-13 [105]. Two studies have assessed the safety, efficacy, and tolerability of dupilumab [106,107]. One of the studies has resulted and has noted reduced COPD exacerbation rates (rate ratio 0.70, 95% CI 0.58–0.86) and, interestingly, improved lung function, lesser respiratory symptoms, and health-related quality of life [107]. This study used a higher cut-off value for the blood eosinophil levels of 300/microL when screening patients. The most common adverse effects were nasopharyngitis, upper respiratory tract infection, and headache. The study’s result revealed more consistent data than the studies performed on monoclonal agents against IL-5, which noted mixed results on COPD exacerbations and no effect on lung function, symptom relief, or improvement in quality of life. It is a consideration for patients who continue to experience frequent exacerbations while on these therapies to be tested for potential occult allergic triggers. Figure 1 overviews assessing and managing patients with refractory COPD.

## 5. Surgical Approaches for Refractory COPD Management

Certain motivated patients with advanced or refractory COPD should be evaluated for these invasive management strategies. These options are lung volume reduction surgery (LVRS) via thoracotomy or thoracoscopic approach and non-surgical bronchoscopic lung volume reduction (BLVR) using endobronchial valves (EBV).

The mechanism by which LVRS might provide benefits needs to be clarified. It has been suggested that surgical excision or valve-mediated collapse of the most emphysematous part of the lung tissue may reduce hyperinflation, reduce load, and improve synchrony of the diaphragm and intercostal muscles, returning the diaphragm to a more normal curved configuration, improve cardiac output and ventilation–perfusion matching [108,109,110]. It is also postulated that LVRS improves endothelial function and decreases inflammatory cytokines like tumor necrosis factor-alpha, interleukin-6, and -18 [111,112]. In selected subgroups of patients, LVRS and BLVR only modestly improve spirometry, lung volumes, dyspnea score, exercise capacity, quality of life, and long-term survival [113,114,115,116]. However, patients with refractory or advanced COPD are often frail with multiple medical co-morbidities and are more prone to potential harm from these invasive interventions. Therefore, pre-procedural evaluation at a high-volume center and informed decision making are necessary for adequate patient selection.

### 5.1. Lung Volume Reduction Surgery

The National Emphysema Treatment Trial (NETT) is the largest randomized trial that compared the benefits of LVRS with maximal medical therapy in over 1200 patients with advanced emphysema [117]. Patients underwent a mandatory pulmonary rehabilitation program for 6–10 weeks before randomization. Two-thirds of the patients underwent LVRS via thoracotomy and one-third by video-assisted thoracoscopic surgery (VATS). Short-term (30-day) mortality was higher in the surgical group (2.2%) vs. the medical therapy group (0.2%), with *p* < 0.001. However, the long-term mortality rate (2 years) did not differ. There was a clinically and statistically significant improvement in exercise capacity and lung function, which gradually decreased over five years [117,118,119]. In the subgroup analysis, patients with upper-lobe predominant emphysema and poor exercise tolerance appeared to have a better outcome [117]. Of significant note, there was a greater death rate (16% vs. 0 in the medical therapy group) among the high-risk subgroup of patients. These patients had forced expiratory volume in 1 s FEV1 of 20% or less and either homogenous emphysema or a diffusion capacity for carbon monoxide (DLCO) of 20% or less [120]. These patients were subsequently excluded and contraindicated to undergo LVRS. Subsequently, a Cochrane systematic review of 11 studies including over 1700 patients showed higher short-term mortality in the LVRS group (OR 6.16, 95% CI 3.22–11.79) but favorable long-term mortality for LVRS (OR 0.76, 95% CI 0.61–0.95) [113]. Significantly, more than 70% of patients included in this systematic review were from the NETT trial. LVRS has shown improvement in clinically meaningful outcomes like relief of dyspnea, significant improvement in quality of life, and improvement in oxygenation [119,121]. Major complications of LVRS include significant pulmonary and cardiac morbidity like arrhythmias, prolonged mechanical ventilation, pneumonia, re-intubation, and persistent air leaks [119,122]. The indications and general contraindications for LVRS are described in Table 2 and Table 3, respectively [117].

### 5.2. Bronchoscopic Lung Volume Reduction Surgery (BLVRS)

Various techniques have been developed to treat lung hyperinflation due to emphysema using bronchoscopic approaches in recent years. These techniques include endobronchial valve (EBV) placement, coil placement, sealants, and thermal airway ablation. Endobronchial valve placements have received widespread regulatory approval; the rest are currently in the investigational stages. The rationale is essentially the same as in LVRS, but is achieved using a less invasive bronchoscopic approach and typically has less stringent selection criteria than LVRS. The two currently available valve systems are the zephyr duckbill and spiration umbrella valves [123,124]. One major consideration factor is the determination of fissure integrity to prevent collateral ventilation. If the fissure is incomplete or the completeness score is low, then success with BLVRS/EBV is unlikely, and a surgical approach should be explored [125,126]. Trials have demonstrated that EBV therapy can improve lung function, quality of line, and exercise tolerance, like LVRS. Longer-term follow-up also suggests modest survival benefits [127]. A common short-term complication of EBV placement is pneumothorax, which can be seen in 20–30% of patients [128]. Other acute complications include hypoxemia, central airway distortion, hemoptysis, pneumonia, and exacerbation of COPD [129]. Long-term complications include forming granulation tissue, challenging valve removal, valve malfunction or migration, bacterial colonization, or infection [129].

## 6. Lung Transplantation

COPD is the most common reason for a lung transplant. Over the last decade, there have been an increasing number of lung transplants performed for idiopathic pulmonary fibrosis (IPF) [130]. Lung transplant aims to improve survival and quality of life. Given the limited availability of organs, improving survival is the priority for patient selection [131]. The decision to proceed with lung transplantation is usually complex. It requires referral to a transplant center for shared decision making with the patient and family and many tests and investigations to ensure safe and appropriate candidacy [132]. Multiple studies have shown improved functional outcomes, such as increased exercise capacity, improved dyspnea score, and enhanced spirometry data [133,134]. The International Society for Heart and Lung Transplantation (ISHLT) shows a median survival of 7.1 years for patients undergoing lung transplantation, which most recently increased to 8.3 years [135]. Typical indications include patients with progressive dyspnea despite maximal medical management, pulmonary rehabilitation, and oxygen therapy who are not candidates for lung volume reduction surgery. The BODE index determines patients who carry high mortality (patients with FEV1 < 25% of predicted and those with resting hypoxemia and hypercapnia defined by PaO_2_ < 60 mm Hg and PaCO_2_ > 50 mm Hg, respectively) [132]. Patients need to have strong social support, be willing to undergo significant surgery, and have frequent follow-up visits post-transplant. Previous LVRS does not negatively impact post-transplant survival [136]. Some of the common contraindications include the following: age > 70 years, body mass index > 35, active smoking or polysubstance abuse, chronic kidney disease, chronic liver failure, severe coronary artery or cerebrovascular disease, and overall frailty [131,132]. Table 4 lists the criteria and contraindications for lung transplantation in patients with advanced COPD.

## 7. Palliative Care

Patients with refractory COPD can achieve improvement in exertional dyspnea, as noted in a few studies using immediate sustained-release morphine or dihydrocodeine [137,138,139]. Moreover, a meta-analysis also emphasized the effectiveness of both systemic and nebulized opioids in dyspnea management [140]. However, three studies from 2020 did not reveal any significant change in dyspnea with the use of sustained-release morphine and oxycodone [141,142,143]. Interestingly, two of these studies used immediate-release morphine on an as-needed basis in the placebo group, making it difficult to assess the isolated effect of sustained-release morphine [141,143]. Another multicenter placebo-controlled trial showed no significant reduction in breathlessness intensity with daily use of low-dose extended-release morphine (8 mg/16 mg per day) over three weeks [144]. No placebo-controlled RCTs have been performed to assess the effect of the transdermal fentanyl patch. Therefore, the use of long-acting opioids for relieving breathlessness is not supported as a general census and is only used for palliation purposes in refractory COPD. A multidisciplinary approach can help refractory COPD patients manage their dyspnea, which includes exercise training (limb endurance, neuroelectric muscle stimulation), accommodation (frequent rests), breathing techniques (diaphragmatic, pursed-lips breathing), distraction (cognitive behavioral therapy, music), and chest wall vibration (leading to stimulation of afferent nerves) [145]. Refractory COPD patients frequently suffer from chronic cough, which can disrupt their activities of daily living. Centrally acting medications like opioids, gabapentin, and pregabalin, as well as peripherally-acting benzonatate, are helpful [146,147]. 

Anxiety and depression usually co-exist in this patient population, and underlying anxiety can cause dyspnea to appear worse. Respiratory stimulants do not assist in improving dyspnea. Cognitive behavior therapy and antidepressant medication use have improved quality of life and reduced mortality risk with referral to mental health specialty care [148,149,150,151].

The BODE index is a multidimensional index that can predict mortality and risk of hospitalization [152]. The desire for invasive care should be explored in patients with advanced COPD who require frequent hospitalizations for severe exacerbations or with debilitating dyspnea, cough, and poor quality of life. Hospice care should be offered to these patients at the end of their lives, and palliative measures to decrease patient suffering should become the focus of treatment.

## 8. Future Directions

Historically, the management of COPD has revolved chiefly around inhaled bronchodilator therapy for maintenance and corticosteroid use during exacerbations. COPD management has not seen significant advancement in years, but this has recently changed [153]. The expanding role of biologics in COPD is on its way. We now understand COPD in terms of phenotypes, which will drive personalized and precision medicine in managing these patients [154]. Stem cell therapy has been studied to manage COPD, but it is far from being ready for clinical application [155]. Technology has been advancing at a rapid pace. Telemedicine has been used in COPD management to reduce readmissions. Artificial intelligence will be the next leap in COPD management. AI can play a vital role in understanding COPD heterogeneity, heart and lung interactions, and developing personalized management algorithms [156]. In the field of biologics, IL-33 is increasingly being studied as a therapeutic option in COPD. High expression of IL-33 has been observed in patients with COPD, but their role in pathogenesis is still unclear. Anti-IL-33 in animal models has been shown to reduce lung inflammation [157]. Currently, there are three major phase III clinical trials ongoing to study the role of anti-IL-33 agent tozorakimab in COPD patients (OBERON trial, NCT05166889; TITANIA trial, NCT05158387; PROSPERO trial, NCT05742802). Table 5 lists a few recent trials studying moderate to severe COPD therapeutic options.

## 9. Conclusions

COPD is a common condition and carries a significant socio-economic burden around the globe. Refractory COPD includes a cohort of patients who remain symptomatic despite achieving maximal guideline-recommended medical therapy, including the use of supplemental oxygen and pulmonary rehabilitation. These patients should be managed using a multidimensional approach. In addition to ensuring the correct inhaler technique, clinicians should look out for other common co-morbidities like CAD, CVA, anxiety, and depression. These patients also have concomitant pulmonary disorders like asthma overlap, malignancy, interstitial lung disease (ILD), and bronchiectasis, which should be evaluated and managed accordingly with the goal of symptomatic improvement. Emphysema-predominant phenotype patients should be considered for bronchoscopic or surgical lung volume reduction. A bronchitis phenotype should be considered for chronic antibiotic or PDE-4 inhibitor therapy after shared decision making with the patient regarding side effects. Lung transplantation should always be considered in patients with advanced COPD, with no contraindications, and who are willing to undergo an organ transplant. This involves early referral to a lung transplant center and shared decision making with the patient and family members if involved. Timely consideration of palliative care is essential to improve quality of life and symptom management. The role of anti-IL five therapy is still being explored, and currently, patients with COPD–asthma overlap with eosinophilia are potential candidates. Further research is needed to find targeted therapies in patients with refractory COPD and optimize treatment approaches by potentially integrating artificial intelligence and deep machine learning to provide personalized treatment to these challenging patients.

## Figures and Tables

**Table 1:** Description of recent studies examining the efficacy of home non-invasive positive pressure ventilation in chronic advanced obstructive pulmonary disease patients.

Author (Year)	Type of Study	Patients	Group 1	Group 2	Outcomes
Murphy et al. (2017) [45]	Randomized controlled trial	116 patients with persistent hypercapnia (PaCO_2_ > 53 mm Hg) 2–4 weeks post COPD exacerbation	NIPPV plus oxygen therapy	Oxygen therapy alone	Median time to readmission lower in NIPPV group (4.3 months vs. 1.4 months; HR 0.49; *p* = 0.002). 17% absolute risk reduction in 12-month mortality or readmission rate in NIPPV group
Kohnlein et al. (2014) [50]	Randomized controlled trial	195 patients with advanced COPD with PaCO_2_ > 52 mm Hg and pH > 7.35. NIPPV was targeted to reduce baseline PaCO_2_ by at least 20%.	NIPPV plus standard medical therapy	Standard medical therapy alone	Lower 1-year mortality in the NIPPV group (12% vs. 33%; HR 0.24 (95% CI 0.11–0.49; *p* = 0.0004)
McEvoy et al. (2009) [51]	Randomized controlled trial	144 patients with severe oxygen dependent COPD and PaCO_2_ > 46 mm Hg	NIPPV plus LTOT	LTOT alone	Lower mortality was observed in NIPPV group (HR 0.63, 95% CI 0.40 to 0.99, *p* = 0.045) but had worsening quality of life.
Clini et al. (2002) [52]	Randomized controlled trial	90 patients with severe oxygen depended COPD were used to assess NIPPV impact on QOL and resource utilization.	NIPPV plus LTOT	LTOT alone	At 2 years, no difference in mortality and hospital readmission. NIPPV group although noted significant decrease in ICU admission and improved HRQOL scores.
Nagata et al. (2022) [53]	Randomized controlled trial	104 patients with severe oxygen dependent COPD and daytime hypercapnia used to assess efficacy of HFNC in reducing exacerbations	HFNC	LTOT with low flow/regular oxygen	Significant reduction in episodes and prolonged duration without acute exacerbations in HFNC group. HFNC group also showed improved HRQoL scores, PFT parameters, and peripheral oxygen saturation.

Abbreviations: NIPPV—non-invasive positive pressure ventilation; PaCO_2_—partial pressure of arterial carbon dioxide; COPD—chronic obstructive pulmonary disease; LTOT—long-term oxygen therapy; HFNC—high-flow nasal cannula; HRQoL—health-related quality of life; PFT—pulmonary function tests; HR—hazard ratio; QoL—quality of life.

**Table 2:** Description of indication/inclusion criteria for patients considered for lung volume reduction surgery (LVRS) and bronchoscopic lung volume reduction surgery (BLVRS).

Parameters	Lung Volume Reduction Surgery	Bronchoscopic Lung Volume Reduction Surgery
Clinical	Age < 75 years	No typical age cut-off
	Quit smoking > 6 months.	Quit smoking > 4 months
	Clinical exam indicative of emphysema	Clinical exam indicative of emphysema
	Uncontrolled symptoms despite maximal medical management and pulmonary rehabilitation	Symptomatic despite maximal medical therapy (stable on <20 mg prednisone or equivalent/day)
	BMI < 40 kg/m^2^	BMI < 35 kg/m^2^
Physiological	Post-bronchodilator FEV1 < 45% of predicted	FEV1 15–45% of predicted
	TLC > 100% of predicted, RV > 150% of predicted indicating hyperinflation	TLC > 100% of predicted. RV > 175% of predicted
	Post-pulmonary rehabilitation 6MWD > 140 m	6MWD 100–500 m
Imaging	CT chest confirming severe emphysema, ideally upper lobe predominant	CT chest confirming emphysema (could be homogenous)
		Little to no collateral ventilation of the targeted lobe

Abbreviations: FEV—forced expiratory volume; TLC—total lung capacity; RV—residual volume; 6MWD—six-minute walk distance; BMI—body mass index; CT—computed tomography.

**Table 3:** Description of contraindication/exclusion criteria for patients considered for lung volume reduction surgery (LVRS) and bronchoscopic lung volume reduction surgery (BLVRS).

Type of Procedure	General Contraindications/Exclusion Criteria
Lung volume reduction surgery (LVRS)	Age > 75 years
	Active smoker
	Previous thoracic surgeries/procedures, chest wall deformity
	Pulmonary hypertension
	Clinically significant bronchiectasis
	Significant cardiac co-morbidities like heart failure (LVEF < 45%), uncontrolled hypertension, myocardial infarction
	FEV1 < 20% of predicted with either DLCO < 20% of predicted or homogenous emphysema
	Severe hypercapnia PaCO_2_ > 60 mm Hg
	Severe hypoxemia PaO_2_ < 45 mm Hg
	Significant pleuro-parenchymal interstitial lung disease
Bronchoscopic lung volume reduction surgery (BLVRS)	Active pulmonary infection/pneumonia
	Large bullae involving > 30% of either lung
	Severe hypercapnia PaCO_2_ > 60 mm Hg
	Severe hypoxemia PaO_2_ < 45 mm Hg
	Prior lung transplant, LVRS, median sternotomy, lobectomy
	Significant cardiac co-morbidities like heart failure (LVEF < 45%), unstable cardiac arrhythmia, MI, CVA

Abbreviations: LVEF—left ventricular ejection fraction; FEV—forced expiratory volume; PaCO_2_—partial pressure of arterial carbon dioxide; PaO_2_—partial pressure of arterial oxygen; MI—myocardial infarction; CVA—cerebrovascular accident.

**Table 4:** Lung transplantation criteria and contraindications in patients with advanced chronic obstructive pulmonary disease.

Major Criteria for Lung Transplantation
Advanced lung disease despite maximal medical management, including pulmonary rehabilitation and oxygen therapy if indicated
Lack of candidacy for lung volume reduction surgery (LVRS)
Post-bronchodilator FEV1 < 25% of predicted
Resting hypercapnia with PaCO_2_ > 50 mm Hg or hypoxemia with PaO_2_ < 60 mm Hg
Body mass index (BMI), airflow obstruction, dyspnea, and exercise capacity (BODE) index score ≥ 5
**Relative Contraindications**
Advanced age (>70 years old)
Active tobacco use
Poor functional status, unable to participated in pulmonary rehabilitation
Frailty, lack of social support at home
Severe osteopenia or osteoporosis
Severe co-morbidities like cirrhosis or advanced chronic kidney disease
Class II obesity and higher (BMI > 35) or underweight (BMI < 16)

Abbreviations: FEV—forced expiratory volume; PaCO_2_—partial pressure of arterial carbon dioxide; PaO_2_—partial pressure of arterial oxygen; BMI—body mass index.

**Table 5:** Recent clinical trials studying moderate to severe COPD therapeutic options.

Clinical Trial	Type of Study	Intervention Group	Control Group	Primary Outcomes	Adverse Events
NCT04072887	Interventional randomized phase 2 trial	Oral QBW251 (icenticaftor) at varying dosing in patients with COPD on triple therapy	COPD patients on triple therapy	No change in FEV1 after 12 weeks in the intervention group but had reduced cough, sputum, and rescue inhaler use	All treatments were well tolerated
NCT04535986	Interventional randomized phase 3 trial	Nebulized ensifentrine twice daily for 24 or 48 weeks in patients with moderate to severe COPD	Placebo twice daily	Intervention group had more improvement in FEV1 and dyspnea scores	No difference in adverse events
NCT03937479	Interventional randomized parallel group phase 2b trial	Varying doses of nebulized ensifentrine twice daily in addition to tiotropium in moderate to severe COPD patients	Placebo twice daily in addition to tiotropium in moderate to severe COPD patients	Intervention group at all doses superior in terms of improvement in FEV1 and dyspnea scores	No difference in adverse events

## Data Availability

Not applicable.
